# Sex differences in resting-state functional networks in awake rats

**DOI:** 10.21203/rs.3.rs-2684325/v1

**Published:** 2023-03-16

**Authors:** Qiong Li, Nanyin Zhang

**Affiliations:** The Pennsylvania State University; The Pennsylvania State University

**Keywords:** sex differences, resting state, fMRI, awake, rat

## Abstract

Sex-related differences can be found in many brain disorders and psychophysiological traits, highlighting the importance to systematically understand the sex differences in brain function in humans and animal models. Despite emerging effort to address sex differences in behaviors and disease models in rodents, how brain-wide functional connectivity (FC) patterns differ between male and female rats remains largely unknown. Here we used resting-state functional magnetic resonance imaging (rsfMRI) to investigate regional and systems-level differences between female and male rats. Our data show that female rats display stronger hypothalamus connectivity, whereas male rats exhibit more prominent striatum-related connectivity. At the global scale, female rats demonstrate stronger segregation within the cortical and subcortical systems, while male rats display more prominent cortico-subcortical interactions, particularly between the cortex and striatum. Taken together, these data provide a comprehensive framework of sex differences in resting-state connectivity patterns in the awake rat brain, and offer a reference for studies aiming to reveal sex-related FC differences in different animal models of brain disorders.

## Introduction

Sex-related differences can be observed in heath and disease (Paus, 2010). For instance, women are more likely to develop Alzheimer’s disease, anxiety- and depression-related disorders, while men have a higher prevalence in schizophrenia, Parkinson’s disease and autism (Palanza & Parmigiani, 2017). A key factor contributing/causing the sex dimorphism is sex-dependent differences in brain structure and function. In the brain, sex differences exist in cell morphology (Juraska et al., 1989), neurotransmitters (Bowman et al., 2004; Davis et al., 1999), region-specific brain volume (Spring et al., 2007), developmental trajectory (Qiu et al., 2018) and circuit-behavior correlates (Li et al., 2016). Therefore, a central task to understand sex-specific behavioral patterns and vulnerabilities is to comprehensively reveal sex differences in the brain.

Historically, animal models of brain disorders are built with a disproportional male-female ratio (Cahill & Aswad, 2015; Karp et al., 2017). It has been reported that the number of male animals used is 5.5 times higher than that of female animals in neuroscience research (Beery & Zucker, 2011).The sex imbalance in animal models may have impeded the understanding of mechanisms underlying sex-biased disease (Anderson, 2008). To address this issue, emerging effort have been spent to address sex differences in behaviors and disease models in rodents, such as social interaction (Bredewold & Veenema, 2018; Cox & Rissman, 2011), compulsive behavior (Kokras & Dalla, 2014; Mathis et al., 2011), addiction behavior (Barker et al., 2010; Quigley et al., 2021), stress copying strategy (Bangasser & Cuarenta, 2021; Bangasser & Wicks, 2017; Campbell et al., 2003) and a series of physiological traits (Karp et al., 2017). One potential factor contributing to these sex-related differences could be sex differences in functional connectivity within specific neural pathways and/or bran networks. Nonetheless, exactly how brain-wide functional connectivity (FC) patterns differ between male and female rodents remains unknown.

FC at the whole-brain level can be routinely measured by resting-state functional magnetic resonance imaging (rsfMRI). To elucidate sex differences in resting-state FC (RSFC) and functional network patterns in rodents, here we acquired and analyzed rsfMRI data in female and male rats. All data were obtained at the awake state (Dopfel et al., 2019; Liang et al., 2011; Ma et al., 2022) to avoid potential confounding effects of anesthesia (Gao et al., 2017; Paasonen et al., 2018; Liang et al., 2012; Liang et al., 2015). RSFC quantified at the awake state also allows for linking neural circuit function to behavioral measures (Tu et al., 2021a; Tu et al., 2021b). Our data demonstrate significant sex differences in RSFC in rats. Male rats show stronger RSFC between the cortex and subcortex, whereas female rats demonstrate more prominent local processing within the cortex and subcortex. Interestingly, RSFC in specific brain regions (e.g. striatum and hypothalamus) exhibit strong sex-dependent bias, and can provide high predicting accuracy for sex (> 80%). At the global level, the whole-brain network organization is generally similar between males and females, but higher small-worldness and higher modularity are observed in females.

## Methods And Materials

### Animals

Data were collected from 14 adult female Long-Evans (LE) rats and 21 adult male LE rats. Animals with the same sex were pair housed in standard cages with free access to food and water. Ambient temperature of the housing room was maintained at 22–24°C with a 12h light: 12h dark cycle. All experimental procedures were approved by the Animal Care and Use Committee at the Pennsylvania State University. Animals of both sexes are balanced in age (female: post-natal 88.8 ± 6.0 days (average ± stdev); male: post-natal 89.2 ± 10.2 days). Females had lower weight (245.0 ± 23.9 g (average ± stdev)) than males (383.2.0 ± 33.8 g).

### Experimental procedures

#### Acclimation

Before imaging, all animals were handled for 3–5 min daily for 5 consecutive days before being subjected to the acclimation procedure. Briefly, animals were first anesthetized with isoflurane and then retrained in a restrainer with the nose and teeth secured. After the animal woke up, they were put in a mock scanner with MRI noise being played. Animals were acclimated for 7 days with progressively increasing time: 15 mins on day 1, 30 mins on day 2, 45 minutes on day 3, and 60 minutes on all following days. This procedure is shown to habituate the animal to the restrainer and scanning environment by reducing animals’ stress and motion. Details of this procedure can be found in (Dopfel & Zhang, 2018). The restrainer design used in the present study is shown in Fig. S1.

#### fMRI

Animals were briefly anesthetized with isoflurane and then placed into the restrainer (Fig. S1) integrated with a 3-channel surface coil for MR signal receiving (RAPID MR International, Columbus, OH) and a birdcage coil for signal transmission (Bruker Billerica, MA, USA). All imaging sessions were conducted on a 7T Bruker 70/30 Biospec MRI scanner running ParaVision 6.0.1. All rsfMRI data were collected when animals were fully awake using a T2*-weighted echo-planar-imaging (EPI) sequence in the axial orientation (no acceleration). The parameters of rsfMRI scans are: repetition time (TR) = 1000 ms; echo time (TE) = 15 ms; field of view (FOV) = 3.2 × 3.2 cm^2^; slice number = 20; matrix size = 64×64; thickness = 1mm; flip angle = 60. For each rsfMRI run, 600 volumes were collected, and 2–3 runs were repeated for each imaging session. For each rat, 1–2 sessions were performed. In total, 46 scans were obtained for female rats and 54 scans were obtained for male rats.

#### Estrus phase measurement

To determine the potential effects of the estrus phase in female rats on RSFC, the estrus phase was measured for each rat on the day of scanning. After each imaging session, the vaginal cells were flushed out using 20 ml of saline, and cells were manually labeled under a microscope using the criteria described in (Ajayi & Akhigbe, 2020). In total, 19 estrus phase samples were obtained from 14 female animals.

### Data analysis

#### rsfMRI data processing

rsfMRI data were preprocessed using in-house developed MATLAB scripts (Liu et al., 2020). First, we discarded volumes with excessive motion based on the measure of framewise displacement (FD), which is estimated using the method described in (Power et al., 2012), but using the size of the rat brain. FD was calculated by performing the rigid-body transformation for each volume, which provides six degrees of transformation parameters, including the translation distances in the three orthogonal axes (*xi*, *yi*, *zi*) and rotation angles around the three axes (*αi*, *βi*, *γi*). FD was calculated based on the equation: *FD* = |*Δxi*| + |*Δyi*| + |*Δzi*| + *r* × (| *Δαi*| + |*Δβi*| + |*Δγi*|), where r is the radius of the rat head, estimated at ~ 5 mm using distance from the cortex to the center of the rat head (Ma et al., 2018b; Ma and Zhang, 2018). Volumes with FD > 0.2 mm, along with their adjacent volumes, were removed. In addition, to make sure rsfMRI data were collected at steady-state magnetization, the first 10 volumes of each rsfMRI scan were discarded. Scans with > 15% volumes removed were left out from further analysis. FD was also compared between male and female rats.

After motion scrubbing, the first volume of each rsfMRI scan was manually registered to the corresponding anatomical image with rigid-body transformation using our in-house MATLAB scripts (Liu et al., 2020). Motion correction was then conducted using SPM12. Next, independent component analysis (ICA) cleaning was carried out on each motion-corrected rsfMRI scan (independent component (IC) number = 50). Each IC was manually classified as either a signal or noise component based on their spatiotemporal features, as described in (Liu et al., 2020; Stephen M. Smith et al., 2013). Nuisance signals from the white matter (WM), cerebral spinal fluid (CSF), and motion parameters were voxelwise regressed out from BOLD time series. In addition, unique components of noise ICs were regressed out using soft regression. In this way, only unique parts across regressors are removed but shared parts are maintained (Griffanti et al., 2014). This regression method can avoid the effect of ‘over regression’ when multiple regressors are involved. Prior to the nuisance regression step, we manually labeled voxels in the WM and CSF on the male and female EPI templates, obtained by averaging EPI volumes across female and male rats, respectively, to ensure precise estimation of nuisance signals in males and females (Spring et al., 2007). Finally, rsfMRI images were spatially smoothed (Gaussian kernel, FWHM = 1 mm) and temporally filtered by a 0.01–0.1 Hz bandpass filter.

To account for different sizes of the male and female brain, for each sex group the brain atlas was carefully wrapped onto the corresponding EPI template using 3D slicer (Fedorov et al., 2012) with the reference of anatomical landmarks on each slice, providing sex-specific atlas (shown in Fig. S2). Based on the anatomic definition, the brain was parcellated into 64 bilateral regions of interest (ROIs). The time course of each ROI was obtained by averaging the time courses of all voxels in the ROI. RSFC between ROIs was calculated using Pearson correlation coefficient between their time courses, which was then converted to z score using Fisher’s Z transform. Sex differences in RSFC were determined by two-sample t-tests using a linear mix model with the sex as the fixed effect and the rat as the random effect. The threshold of statistical significance was set at p < 0.05, false discovery rate (FDR) corrected. Our main results were reported using this brain parcellation scheme. However, we also parcellated the brain into 128 unilateral ROIs, and the group-level sex differences in RSFC between these 128 unilateral ROIs were obtained using the same processing methods and reported in Fig. S3.

#### Sex classification

Sex classification based on the brain-wide connectivity pattern of each region was conducted using a nonlinear support vector machine (SVM) (Noble, 2006). SVM can learn the relationships between the input features (i.e. the region’s connectivity pattern) and the outcome (i.e. the sex) via an optimized function to achieve the highest prediction accuracy. For each region, the SVM and a radial basis function (RBF) were applied to train the classification model with a 10-fold cross validation. That is, all scans were randomly separated to the training group and test group with a 9 – 1 ratio (i.e. 90% of scans were assigned to the training group and the other 10% of scans were assigned to the test group). The classification error was obtained using the “kfoldloss” function. This procedure was repeated 5 times for each region and the averaged accuracy rate was obtained. To determine the statistical significance of each prediction, we compared the results with the null distribution based on permutation tests. For each brain region, the null distribution of the accuracy rate was generated by conducting the SVM and cross-validation algorism on permutated data across sexes, and this procedure was repeated 1000 times. The significance of the p-value obtained for the region was then FDR corrected across all 64 regions. The trained model was also independently tested on a separate rsfMRI database published by our group (Fig. S4) (Liu et al., 2020)

#### Hierarchical clustering

Hierarchical clustering was used to examine the whole-brain network organization in males and females. We used a bottom-up clustering method that merges separative components into larger clusters successively. In our study, regions were hierarchically clustered together based on the similarity of connectivity patterns using the Ward’s method (Murtagh & Legendre, 2014; Thirion et al., 2014; Ward Jr, 1963). This method is known to give a accurate and stable clustering pattern for fMRI data (Thirion et al., 2014) by merging two clusters if they have the smallest sum of squared differences between their RSFC patterns. With an empirical threshold, we cut off the dendrogram into 7 clusters.

#### Graph theory analysis

Graph theory analysis was used to evaluate the topological differences of the whole-brain networks between males and females. The RSFC matrix of each scan was binarized based on the graph density (range: 0.1–0.3, step size = 0.05). Global efficiency, small worldness, modularity and assortativity were quantified using the toolbox published in (Rubinov & Sporns, 2010) to examine the network integration, small world feature, segmentation and resilience, respectively. We also compared the degree of each node between males and females.

#### Estrus phase measurement

All imaging data acquired in female rats were divided into the estrus/proestrus (n = 10) and non-estrus (n = 9) subgroups (Dumais et al., 2013), and the potential impact of the estrus phase was estimated by calculating the RSFC differences between the two subgroups.

All data processing was conducted using MATLAB (The Mathworks Inc., Natick, MA, USA).

## Results

### Male and female rats exhibit characteristic RSFC differences between brain regions

**Figure 1:**
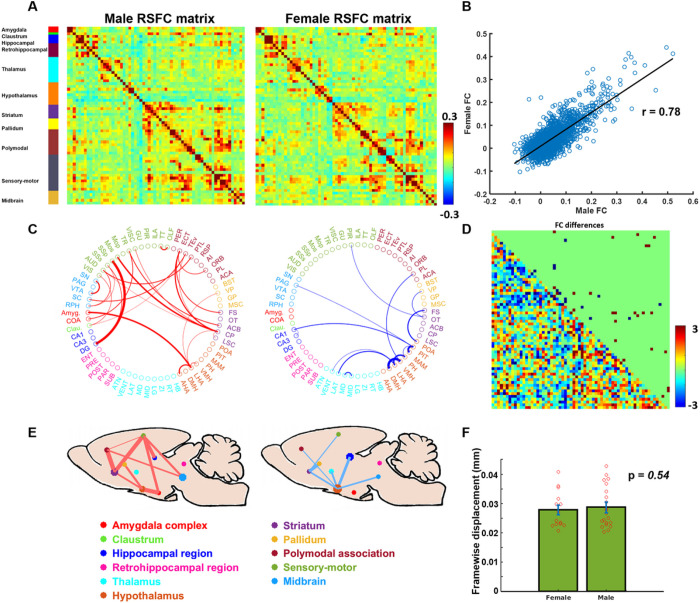
Whole-brain RSFC patterns in males and females. (A) Left, averaged matrix of connectivity between 64 bilateral ROIs in males. Right, averaged matrix connectivity between 64 bilateral ROIs in females. (B) Entry-to-entry correlation between male and female RSFC matrices (r = 0.78, p < 10^−10^). (C) Circular graph showing significant sex differences in FC. Red lines indicate stronger connectivity in males and blue lines indicate stronger connectivity in females. The node color indicates the brain system they belong to (see caption in E). (D) The lower triangle shows t scores of RSFC comparison between males and females (linear-mixed model, two-sample t-tests). The upper triangle shows connections displaying significant RSFC differences (p<0.05 after FDR correction). (E) Connections showing significant sex differences in RSFC, organized into 10 brain systems and overlaid on a sagittal rat brain. The node size is proportional to the percentage of the total number of significantly changed within-system connections relative to the total number of all possible within-system connections, and the edge width is proportional to the percentage of the total number of significantly changed connections between two systems relative to the total number of all possible between-system connections. Left, connections with stronger RSFC in males. Right, connections with stronger RSFC in females. (F) Motion levels in males and females, quantified by the averaged FD of rsfMRI scans. Each dot represents the averaged FD of each animal.

We first examined sex differences in RSFC across brain-wide connections. The whole brain was parcellated into 64 bilateral ROIs. Figure 1A shows RSFC matrices, displaying pairwise RSFC between every two ROIs, in males and females. Overall, the whole-brain connectivity structure is consistent between males and females, reflected by a high similarity between the two RSFC matrices. The entry-to-entry correlation between the male and female RSFC matrices was 0.78 (p < 10^−10^, Fig. 1B).

Despite the overall consistent whole-brain connectivity pattern, male and female rats still exhibit RSFC differences in a group of connections (linear mixed model, two-sample t-tests, p < 0.05, FDR corrected, Fig. 1C–E). When grouping brain regions involved in these connections, one can observe a clear pattern of sex dimorphism in RSFC. Specifically, male rats display stronger connectivity between the cortex and subcortex, particularly in the cortico-striatal connections. In contrast, female rats show stronger connectivity in the hypothalamus (Fig. 1C–E). Notably, the RSFC differences we observed was not attributed to difference in motion during imaging, as there was no significant difference in the motion level, quantified by the FD, between males and females (Fig. 1F, p = 0.54). In addition, the averaged FD (~0.028 mm, Fig. 1F) was very low relative to the voxel size (0.5 mm), indicating that motion was well controlled in this experiment.

### Different brain regions show heterogeneous predicting power for sex

**Figure 2:**
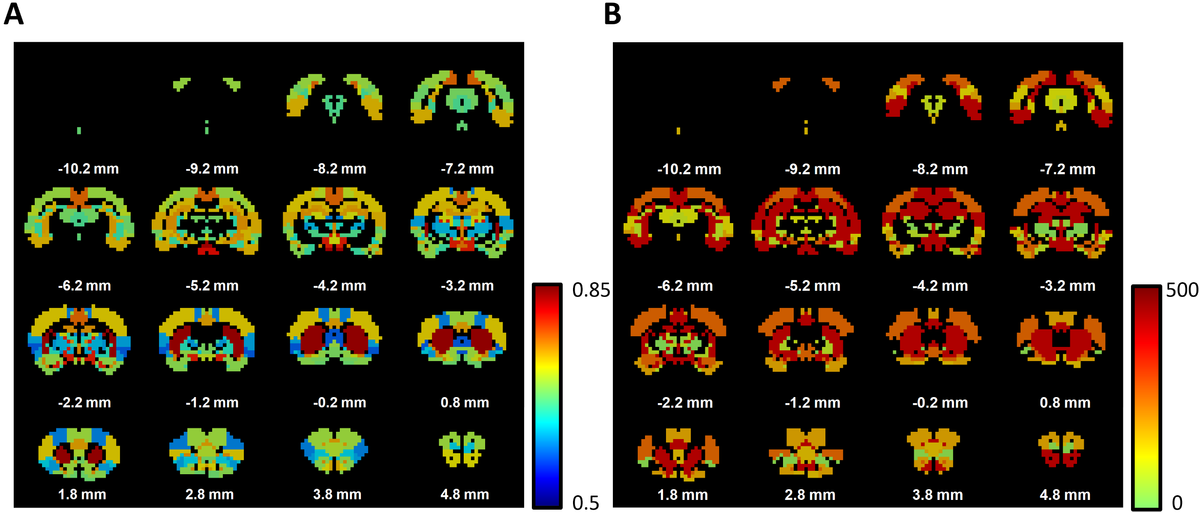
Sex classification based on the RSFC pattern of each brain region. (A) Sex prediction rate. (B) The significance levels of 57 regions exhibiting significant prediction rate (p value < 0.05 after FDR correction). Color indicates the significance level, presented as the inverse of the FDR-corrected p value.

Given that separate brain regions exhibit characteristic sex differences in RSFC, we ask to what extent the RSFC pattern of a specific brain region can be used to predict the sex. To this end, we performed sex classification by employing a support vector machine (SVM) method based on the RSFC pattern of each region (see Methods and Materials for detail) (Steardo Jr et al., 2020; Wang et al., 2019; Wang et al., 2007). Our data indicate that the striatum, hypothalamus, and retrosplenial cortex have the highest predicting power for sex. Specifically, the caudate-putamen (CPu) in the striatum has the highest prediction accuracy rate of 83.2%, based solely on its connectivity with the rest of the brain. Similarly, the hypothalamic regions such as the lateral hypothalamic nucleus, pituitary, and mammillary bodies display a prediction rate of 79.4%, 80.2%, and 82.6%, respectively, and the retrosplenial cortex shows a prediction rate of 76.6%. Interestingly, the vast majority of brain region (57 out of 64) exhibit a statistically significant prediction rate (p < 0.05, FDR corrected) that is above the random level using a permutation test, indicating that there is a sex effect on the RSFC in most brain regions. The prediction rates from all 64 brain regions are shown in Fig. 2A (range: 61.4%–83.2%, mean ± std = 70.2% ± 4.9%), with the significance level shown in Fig. 2B.

We separately tested the prediction power of the SVM model on an independent open database our lab published (Liu et al., 2020). Consistent with our testing results on the current cohort of animals, we found that the hypothalamus showed a high level of prediction accuracy rate (Fig. S4, anterior hypothalamic nucleus: 86.85% and lateral hypothalamic area: 78.85%). However, on average the prediction rate on the independent cohort was appreciably lower than the results of the present study. This is most likely due to the fact that different coils were used for the data acquisition between the two datasets (a 3-channel surface coil was used in the present study, whereas a birdcage coil was used for the open database), which affect both the spatial profile and sensitivity of EPI images. In addition, all rats are male for the open database, making it difficult to re-train the SVM model with the dataset.

### Global brain network organization is characterized by prominent cortical-subcortical interactions in males, but more segregated within-cortical and within-subcortical processing in females

**Figure 3:**
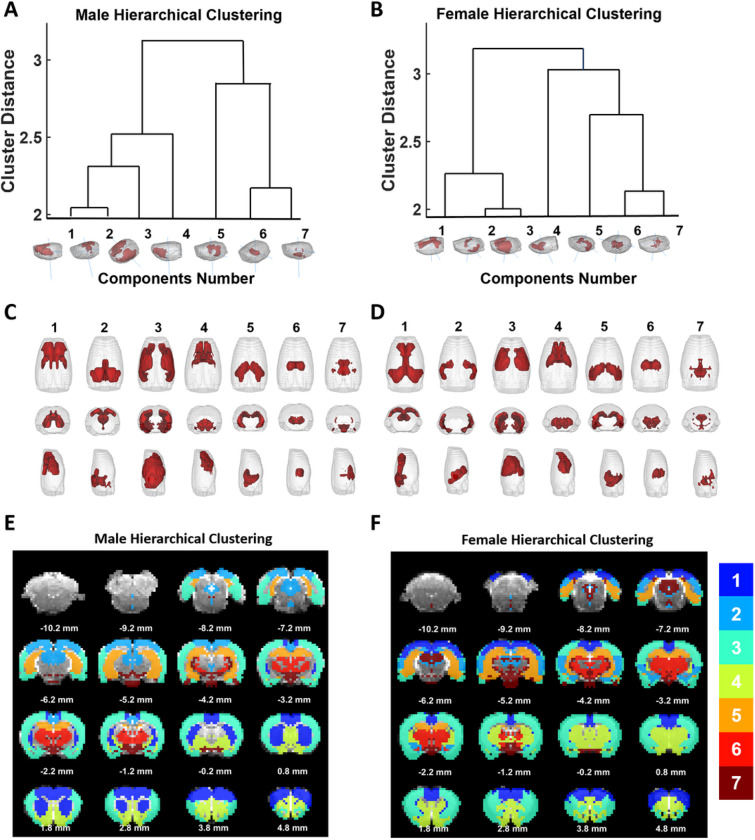
Hierarchy clustering of global brain networks. (A) Hierarchical clustering dendrogram across all brain regions in males. (B) Hierarchical clustering dendrogram in females. (C) 7 clusters in males shown in a 3D view, overlaid on a transparent brain. (D) 7 clusters in females shown in a 3D view, overlaid on a transparent brain. E) 7 clusters in males shown in a slice-by-slice view F) 7 clusters in females shown in a slice-by-slice view.

We further examined the whole-brain network organization between male and female rats using hierarchical clustering (Fig. 3). For each sex, 7 clusters were identified based on an empirical threshold (Fig. 3A–3B). Overall, both males and females exhibit a consistent global brain network organization. For instance, all striatal and pallidal regions are clustered in Cluster 4. Cluster 5 includes the hippocampal and retrohippocampal ROIs. All thalamic nuclei are grouped in Cluster 6, and all hypothalamic nuclei are grouped in Cluster 7 (Fig. 3C–3F). Despite similar organization in subcortical regions, cortical regions are organized in a somewhat different manner between males and females. In males, the anterior cingulate cortex (ACC) and retrosplenial cortex belong to two separate clusters, with the ACC more tightly related to CPu, and the retrosplenial cortex linked to the midbrain area (Fig. 3C, 3E). In contrast, the ACC and retrosplenial cortex are grouped in the same cluster without involving any subcortical or midbrain regions in females (Fig. 3D, 3F). Similarly, at the global organizational level, in females more tight relationship can be observed within cortical clusters (Clusters 1–3) and within subcortical clusters (Cluster 4–7), but cortical and subcortical clusters less related, evidenced by the farthest distance between these two groups of clusters (Fig. 3B). However, in males the striatum/pallidum cluster (Cluster 4) is more tightly related to the cortical clusters (Clusters 1–3), but less involved in the subcortical cluster group (Clusters 5–7, Fig. 3A). Taken together, these data indicate that females are characterized by segregated within-cortical and within-subcortical processing, whereas males tend to have more cortico-subcortical, particularly cortico-striatal interactions in the whole brain organization.

To examine whether differences in clustering identified reflect intrinsic spatial organization of functional connectivity between male and female rats, we shuffled rats from either sex group and randomly divided them to two new groups. The same clustering method was repeated on the two control groups (Fig. S5). The results failed to reveal any appreciable differences in clustering patterns between the two control groups, suggesting the differences in clustering identified indeed reflect sex differences at the global network level.

**Figure 4:**
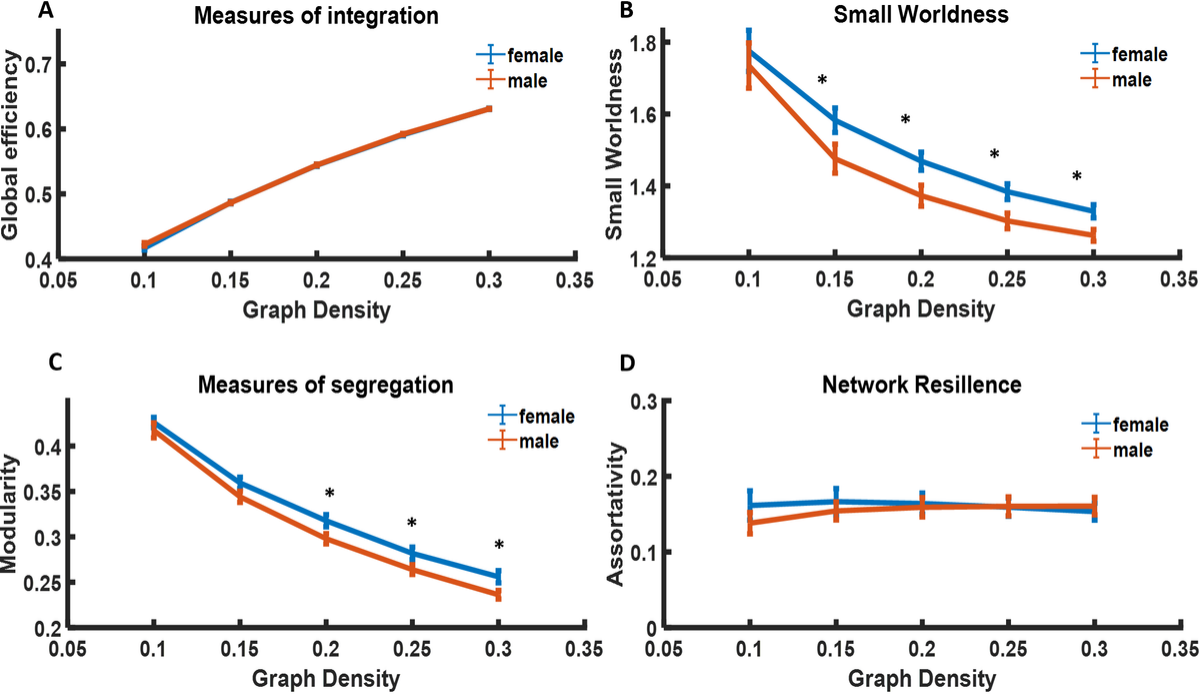
Topological analysis of the whole-brain network in male (red) and female (blue) rats. (A) Global efficiency of both sexes, which measure network integration. (B) Small-worldness of both sexes. (C) Modularity of both sexes, which measures network segregation. (D) Network resilience, measured by assortativity in both sexes. * p<0.05.

Topological analysis of the global brain network suggests that both males and females show similar network integration and network resilience (Fig. 4A&D), consistent with our finding of generally stable network organization between females and males (Fig. 1A&B). However, female rats seem to display higher network segregation (Fig. 4C), which agrees with the notion that information tends to be more locally processed within cortical and subcortical clusters in females (Fig. 3D, 3F). Similar network integration and higher network segregation also lead to higher small worldness in the global brain network in females (Fig. 4B).

**Figure 5:**
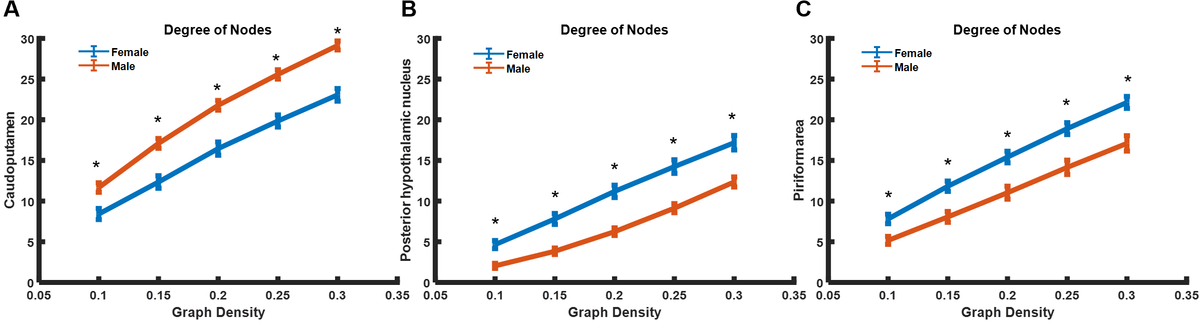
Degree of nodes with significant sex differences. (A) CPu. (B) Posterior hypothalamic nucleus. (C) Piriform area.

Local topological features further illustrate the differences in the centrality of each brain node (Fig. 5). Males exhibit a significantly higher degree in the CPu, whereas females have a significantly higher degree in the piriform cortex and posterior hypothalamus nucleus (p < 0.05, FDR corrected).

### The potential impact of estrus phases on RSFC in female rats

To examine whether estrus phases play a role in RSFC, we compared the RSFC matrices when scanning took place during the estrus/proestrus phases (n = 10) versus the RSFC matrices when scanning took place during the non-estrus phase (n = 9) in female rats. No significant difference in any connection was detected at the statistical significance level of p < 0.05 after FDR correction, likely due to smaller sample sizes in each subgroup. However, two connections, both involving the bed nucleus of the stria terminalis (BNST), indicate a trend of significance (p < 0.001, without FDR correction), indicating that this region might be affected by hormone fluctuation (Fig. S6).

## Discussion

To the best of our knowledge, this is the first study investigating sex-related RSFC differences in individual neural connections and the whole-brain network in awake rats. Our data indicate that individual connections exhibit significant sex differences, with males showing higher cortico-striatal connectivity, and females displaying stronger hypothalamus-related connectivity. To further examine the relative role of specific regions in RSFC sex differences, RSFC patterns of individual brain regions were used to predict sex with a SVM classification method. The striatum, hypothalamus and retrosplenial cortex exhibited the highest predicting power, again demonstrating regional heterogeneity in RSFC sex differences. At the global network level, females showed stronger within-cortical and within-subcortical segregation, whereas males displayed more prominent cortico-subcortical interactions. These data provide a comprehensive framework of sex differences in brain-wide connectivity, and offer a reference for studies aiming to reveal sex-related RSFC differences in animal models of brain disorders.

A prominent region that displays stronger connectivity in females is the hypothalamus. This result well agrees with the finding of sex differences in brain structure (Spring et al., 2007), suggesting that the hypothalamus is different in shape and size between male and female mice. Indeed, the hypothalamus is arguably the most well-documented region exhibiting sex-dimorphism, known to have abundant steroid receptors, and govern mating and stress response (Squire et al., 2012; Yang et al., 2013). In our study, females show stronger connectivity between the hypothalamus and pituitary, consistent with the literature report of sex differences in the hypothalamic-pituitary-adrenal (HPA) axis (Heck & Handa, 2019). As the central system that regulates the stress response, the HPA axis shows sex differences in the neuropeptide regulation (Heck & Handa, 2019). For example, both basal stress hormone and HPA stimulation-evoked stress hormone release are higher in females (Heck & Handa, 2019; Kokras et al., 2019). In addition to the hypothalamus, female rats show stronger connectivity between two olfactory regions—the piriform cortex and olfactory tubercle, and this connection is tightly associated with the pheromone-related psychosexual function (Cherry & Baum, 2020).

In males, a characteristic region showing sex differences in RSFC is the CPu, which is also in line with the previous finding of sex differences in the shape of CPu in mice (Spring et al., 2007). In addition, our data reveal stronger RSFC between the CPu and polymodal association cortex, hypothalamus, and the sensory-motor cortex in male rats, consistent with the report of sex differences in the striatal-cortical and striatal-subcortical circuits (Lei et al., 2016). In a similar vein, the activity of the putamen is positively correlated with the concentration of steroids hormone (Dreher et al., 2007), and males tend to show increased putamen activation than females in reward-related and risk-seeking behaviors (Lighthall et al., 2012). Furthermore, in our data male rats display stronger connectivity between the lateral hypothalamus and amygdala. This circuit is known to be involved in stress response, especially rodents’ avoidance behavior in the context of danger (Dopfel et al., 2019; Weera et al., 2021; Liang et al., 2014). Stronger connectivity in this circuit in males may help elucidate the mechanism underlying differential reactions to potential dangers between males and females.

Based on the finding that multiple brain regions exhibit sex differences in their RSFC patterns, we then tested the possibility to predict sex based on the connectivity patterns of individual regions. Our analysis is different from previous studies on RSFC-based sex classification in humans, which are mostly based on whole-brain connectivity patterns (Stephen M Smith et al., 2013; Zhang et al., 2018). The whole-brain network based method has two drawbacks: First, the high dimensionality of whole-brain connectivity matrices may make the method suffer from the problem of overfitting. Second, results from whole-brain network based classification are more difficult to interpret, as the contributions of specific regions are less straightforward. A recent study used SVM to classify sex in humans based on a single region’s connectome and obtained a stable sex prediction rate both within samples and between samples, with the mean prediction rate of 68.7 (Weis et al., 2020). This result is consistent with our study using a similar method (mean prediction rate ± std = 70.2% ± 4.9%). These data together demonstrate that, even with a relatively small sample size, most brain regions can achieve a significantly higher classification rate than a random guess, indicating sex-dependent effects on these regions. Interestingly, separate regions exhibit pronounced differences in the prediction accuracy, indicating spatially heterogeneous sex differences in the brain. For instance, the olfactory-related and cortical-striatum systems showed higher predication power than other regions, agreeing with the results from human studies (Cherry & Baum, 2020; Lei et al., 2016). It has to be pointed out that the ultimate goal of testing the predicting power of individual regions is not necessarily to predict sex, but to examine whether separate brain regions contain more sex-specific information that can be reflected by their functional connectivity patterns. RSFC-based sex prediction in rats may be trivial by itself, but the difference in the prediction power among brain regions may reflect the underlying sex specific neurobiology, which can be significant.

Sex hormone changes during the estrus phase may also affect RSFC. It was reported that fluctuations of estradiol and progesterone can induce behavior differences in rodents, such as the stress level in a restraining condition (Shansky et al., 2006), and these differences can be reflected in RSFC. In the present study, when comparing the RSFC between the proestrus/estrus and non-estrus groups, we did not observe a profound sex hormone effect in RSFC, consistent with meta-analysis studies demonstrating that data from female rats are not more variable than male rats (Becker et al., 2016; Prendergast et al., 2014). Another possibility is that the effects of short-lived hormonal fluctuations along the menstrual cycle cannot be reflected by the RSFC measure (Van Den Heuvel & Sporns, 2011). However, given the limited sample size in individual estrus phase subgroups, this result should be interpreted with caution.

The significance of the present study can be appreciated from two aspects. First, it provides important insight into understanding the sex-related neurobiology. Compared to human studies, the environmental and genetic background in lab animals are relatively uniform, making it more likely to identify sex-related differences with a relatively small sample size. Furthermore, sex-related differences in humans are not only determined by biological factors but can be compounded by environmental and sociocultural factors (Becker et al., 2017). Second, the knowledge gained in studying sex differences in rodents may further be used to inform human studies. A number of studies have shown conserved sex differences between rodents and humans. For instance, studies suggest addiction-like behavior in both humans and rodents shares similar sex-related differences (Becker et al., 2017; Carroll & Lynch, 2016). Thus, understanding sex differences in preclinical models of brain disorders may lend critical information to future human research, and can also reduce sex-related bias at the preclinical stage.

There are several potential limitations in present study. First, 14 females and 21 males were used, making the sample size slightly unbalanced. However, we believe this unbalanced sample size issue can be largely mitigated by the linear mixed model applied. Second, there is lack of behavioral tests in animals, making it less likely to unambiguously interpret the RSFC sex differences.

In conclusion, in the present study we discovered the sex-related RSFC differences at both the regional and systems-organization level. This study lays the foundation for future studies aiming to reveal sex-related RSFC differences in different animal models of brain disorders and/or studies of age-related sex differences in RSFC (e.g. during development or aging) (Ma et al., 2018a; Ma et al., 2021).
